# Entomological Surveillance Planning Tool (ESPT)-generated actionable evidence on human and vector behaviours optimizes present interventions and reduces exposure to *Anopheles* vectors in two communities of Guna Yala, Panamá

**DOI:** 10.1186/s12936-023-04453-1

**Published:** 2023-01-25

**Authors:** Mario I. Ávila, Élodie A. Vajda, Eileen Jeffrey Gutiérrez, Daragh Gibson, Mariela Mosquera Renteria, Nicolas Presley, Daniel O’Reilly, Allison Tatarsky, Neil F. Lobo

**Affiliations:** 1 Ministerio de Salud de Panamá (MINSA), Panama City, Panama; 2 Malaria Elimination Initiative (MEI), University of California, San Francisco (UCSF), San Francisco, USA; 3 Clinton Health Access Initiative (CHAI), Panama City, Panama; 4 Eck Institute for Global Health, University of Notre Dame (UND), Notre Dame, IN USA

**Keywords:** Bionomics, Exophagic, Human behavior, Gap in protection, Malaria, *Ny. albimanus*

## Abstract

**Background:**

Although most of Panamá is free from malaria, localized foci of transmission persist, including in the Guna Yala region. Government-led entomological surveillance using an Entomological Surveillance Planning Tool (ESPT) sought to answer programmatically relevant questions on local entomological drivers of transmission and gaps in protection to guide local vector control decision-making.

**Methods:**

The ESPT was used to design a sampling plan to answer priority programmatic questions about the appropriateness of Long Lasting Insecticidal Nets (LLINs) and spaces and times where humans remain exposed to *Anopheles* bites (gaps in protection) in the communities of Permé and Puerto Obaldía, Guna Yala. Adult *Anopheles* were sampled at three time points via human landing catches (HLCs) during the rainy and dry seasons (2018/2019). Human behaviour observations (HBOs) were conducted alongside HLCs to examine intervention use, indoor versus outdoor activity, and sleeping patterns. HLC and HBO data were integrated to evaluate HBO-adjusted human biting rate (HBR).

**Results:**

A total of 7,431 adult *Anopheles* were collected across both sites. Of the 450 specimens molecularly confirmed to species-level, 75.5% (n = 340) were confirmed as *Anopheles Nyssorhynchus albimanus*, followed by *Anopheles (Ny.) aquasalis*. *Anopheles* host seeking activity was demonstrated to be primarily exophagic throughout all sampling periods and in both communities. When adjusted with HBOs, exposure to mosquito bites was predominantly indoors and overnight in Permé (Nov, Mar), compared to predominantly outdoors in Puerto Obaldía (Nov, Mar, Jul). Differences in site-specific human-vector exposure profiles were due to contrasting cultural and lifestyle practices between Permé and Puerto Obaldía (possibly partly influenced by the absence of electricity in Permé), and lower LLIN use in Permé. This evidence supported a previously planned LLIN campaign alongside a social behaviour change communication (SBCC) strategy in the Guna Yala Comarca (Jul 2019), which increased LLIN use. In turn, this led to a reduction of indoor exposure to mosquito bites, and a shift to predominant outdoor exposure to mosquito bites.

**Conclusion:**

ESPT-based question-driven planning and the integration of HBOs, intervention, and HLC data generated evidence towards answering the programmatic questions. This evidence enabled the characterization of site-specific human-vector exposure profiles, and the quantification of remaining gaps in protection. These data also provide important insights into remaining gaps in protection that must be addressed to further reduce human exposure to mosquito bites at these sites.

## Background

The global burden of malaria has substantially declined over the last twenty years, and more countries are striving to eliminate the disease. As of 2021, 40 countries have reached elimination, including most recently, China and El Salvador in 2021, and Algeria and Argentina in 2019. Still, advancement towards malaria elimination is stalling in many places around the world. While malaria case incidence dropped by 27% from 2000 to 2015, malaria case incidence dropped by less than 2% from 2015 to 2019, signaling a stalling rate of decline. In the Americas, malaria case incidence declined by 57% between 2000 and 2019. However, the region’s recent progress has been impacted by the drastic increase in malaria in Venezuela; cases in the Americas increased from 35,500 in 2000, to over 467,000 in 2019 [1].

Understanding why and where transmission is persisting, while also ensuring effective vector control and access to diagnostics and treatment, are key to accelerating progress toward malaria elimination [2]. To ensure effective vector control, entomological surveillance serves to monitor disease vector species, their population dynamics, as well as behavioural traits that impact disease transmission and intervention effectiveness over time and space. For national malaria control programmes (NMCPs), entomological intelligence on local vector bionomics through question-driven baseline or routine surveys can provide actionable evidence to guide the appropriate selection, targeting and optimization of malaria interventions. Further, entomological surveillance can also provide valuable insights into the limitations of interventions in place and expected impact of those interventions [3].

Panamá set the goal of eliminating malaria by 2025 using a focused strategy of epidemiological and entomological surveillance and targeted intervention responses in transmission foci [4, 5]. However, *Plasmodium vivax* malaria [6] remains a major source of morbidity and mortality in Panamá’s indigenous territories, the Comarcas, thus challenging the country’s malaria elimination goals [7]. The heaviest burden of malaria is in the Comarca Guna Yala, an autonomous indigenous territory largely inhabited by the Guna people [7]. Although the Guna indigenous group comprises less than 3% of the total population of Panamá, they shoulder about 90% of the country’s malaria burden [8]. The Comarca Guna Yala’s isolated geographic location, language and cultural barriers, and lack of political commitment have been obstacles to effective malaria control in this region [8]. Additionally, local drivers, including an ecotype that supports vector populations [9–11] and open housing, leave communities vulnerable [12]. Parasite importation from bordering countries and internal migration also pose challenges to malaria elimination in Panamá and in the Comarca Guna Yala [13].

In Guna Yala, the predominant malaria vector is *Anopheles (Nyssorhynchus) albimanus* [8], a major malaria vector across Mesoamerica and the Caribbean. This species is usually considered exophagic and zoophilic, biting primarily during the evening but also throughout the night, although its biting behaviour varies across its distribution [4]. Calzada et al. [8] investigated the epidemiological and entomological factors linked to a malaria outbreak in Guna Yala in 2012. Through mosquito surveys in three Guna communities along the coast of Guna Yala, the authors found that *Ny. albimanus* was the most abundant and widespread species, followed by *Anopheles (Anopheles) punctimacula *sensu lato (*s.l.*) and *Anopheles (Ny.) aquasalis.* The authors also found *Ny. albimanus* to be infected with *P. vivax*, the country’s predominant malaria parasite [8]. In 2018 and 2019, MINSA led entomological surveillance activities in several communities across Guna Yala. Adult mosquito collections from two communities of Guna Yala confirmed the predominance of *Ny. albimanus* in the area [14].

MINSA-implemented vector control in Guna Yala is currently centered on routine indoor residual spraying (IRS) with fenitrothion [8] or clothianidin (as of 2019) [15] in targeted areas that are high risk for malaria transmission [8]. Fogging with deltamethrin and permethrin, larviciding with Vectolex (*Bacillus sphaericus*), and community-based environmental management, are applied in response to newly detected cases and outbreaks. In 2019, MINSA and its implementing partners launched a pilot distribution campaign of long-lasting insecticidal nets (LLINs) and long-lasting insecticidal hammock nets (LLIHNs) in selected areas of Guna Yala [16, 17]. While IRS and LLINs are very effective against endophilic and endophagic *Anopheles* mosquitoes [18, 19], circumstantial effectiveness relies on local vector bionomic characteristics [3] and on human intervention compliance [20, 21].

To assess the relevance of current and/or potential interventions on malaria transmission, vector bionomics data should be integrated with human behaviour data. This is to better understand how intervention use and sleeping patterns overlap with intervention functionality [3, 21]. In doing so, spaces and times where and when people are exposed to malaria vectors outside of the protection of current interventions (i.e. gaps in protection) can be identified and quantified. Pinpointing gaps in protection enables NMCPs to better understand the impact of current interventions deployed on malaria transmission, and aid in decision-making around selection, optimization and deployment of present and additional interventions [20, 21].

The Entomological Surveillance Planning Tool (ESPT) [14, 22] is a decision-support tool for designing question-based entomological surveillance activities utilizing minimum essential indicators to facilitate cost effective, locally tailored, and evidence-based vector control. The ESPT enables NMCPs to quantify gaps in protection [23]. In Panama, the ESPT was used to guide the formulation of programmatic questions and to design an entomological surveillance plan that addresses these questions [14]. This paper describes the methods and results of an ESPT-based plan to investigate whether LLINs are an appropriate intervention in Guna Yala and to pinpoint the remaining gaps in protection, based on the integration of adult *Anopheles* bionomics data with HBO data.

## Methods

### Applying the ESPT

The ESPT was piloted in Guna Yala with MINSA in 2018/2019 [14]. The ESPT-based entomological surveillance plan was formulated to address one of MINSA’s priority programme questions: are LLINs an appropriate intervention against *Anopheles* mosquitoes in Guna Yala given local vector and human behaviour? To answer this question, the ESPT was applied to decide on minimum essential entomological indicators, to delineate a sampling design grounded in available capacity, and to provide a framework for data analysis and interpretation of results [14].

### Study sites

Guna Yala is located along the Caribbean coast of northeast Panamá. The Comarca is composed of 300,000 ha of continental forest and 480 km of coastline, bordered by coral reefs and mangroves. The Guna communities grow coconuts and other crops in lands that were previously rainforest and lowlands, which leads to conducive habitat for *Anopheles* species [24]. The mean annual temperature ranges between 26 and 27 °C, while the mean annual relative humidity and rainfall range between 78 and 90%, and 1600–3000 mm, respectively. The dry season runs from mid-December to April, and the wet season stretches from May to mid-December [8, 14].

**Fig. 1:**
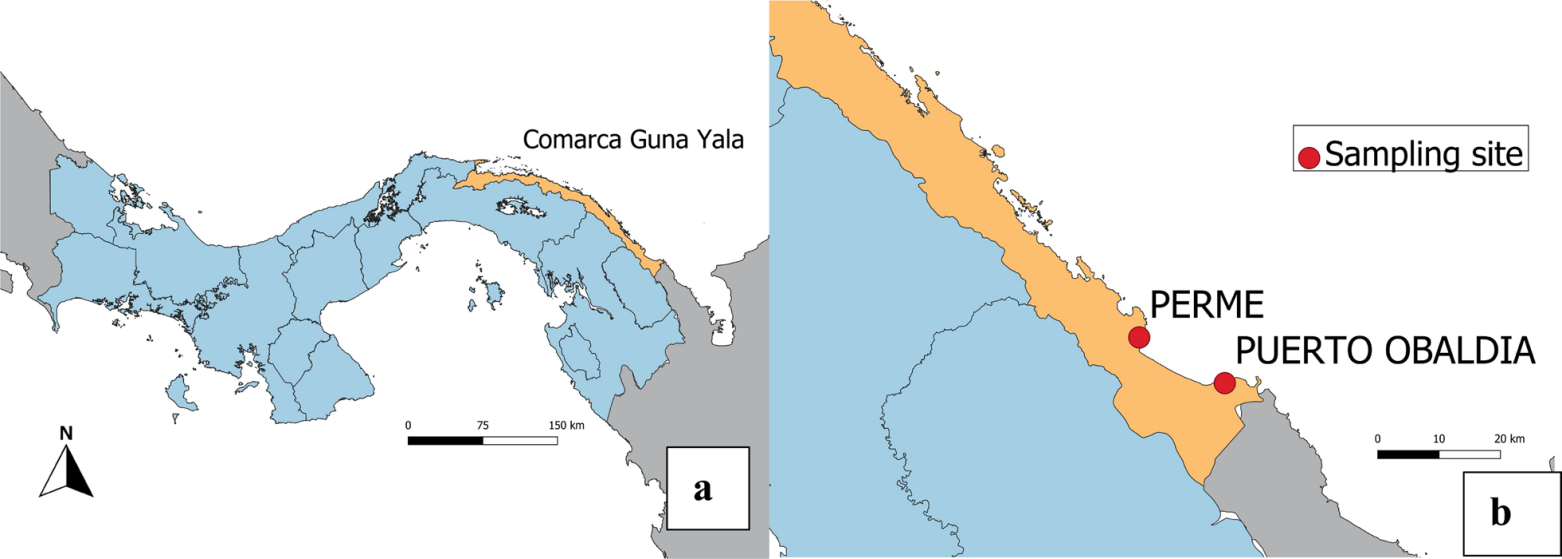
Map of Panama and the entomological sampling sites. **a** Map of Panama. **b** Map of Guna Yala with the two sampling sites

Two sentinel sites were designated for adult mosquito collections: Permé and Puerto Obaldía (Fig. 1). Both are coastal communities flanked with coastal lagoons on one sides and with the edge of the continental forest on the other. Around 20 km along the coastline and 16 km of sea divide Permé from Puerto Obaldía. Permé is a Guna community home to 155 inhabitants, while Puerto Obaldía is primarily an Afro-Latino community counting 596 inhabitants. The communities honor contrasting cultural practices and lifestyles. Permé, has no electricity, and its houses are constructed of thatch rooves, earthen floors, and with walls built of cane sticks secured to posts with natural fibers [8]. In Puerto Obaldía, electricity is present, and houses are typically made with cement/wooden floors and walls, and corrugated iron rooves. Sampling site selection criteria included higher incidence of reported malaria cases and representative eco-epidemiological environments of Guna Yala. In 2018 and 2019, Permé recorded malaria cases year-round, with 30 cases in 2018 and 20 cases in 2019, while Puerto Obaldía recorded 41 cases across 2018, and 12 cases in 2019 (11 cases occurring from January to July, and 1 case in December) [25]. Until 2018, IRS was the primary vector control intervention in both communities. IRS coverage in Permé in 2018 (fenitrothion) and 2019 (clothianidin) was of 85% and 97%. In Puerto Obaldía, IRS coverage was of 97% in both 2018 and 2019, with the same insecticides as applied in Permé. LLINs were distributed in 2019 both communities, and reached a coverage of 99% in Permé and of 89% in Puerto Obaldía [15].

### Entomological sampling

Entomological sampling occurred during three collection periods across different seasons: November 2018 (moderate rainy season), March 2019 (dry season), and July/August 2019 (heavy rainy season). Adult mosquito collections took place sequentially in Permé and Puerto Obaldía. In each site, two sentinel houses typical of local construction were sampled in November, March, and July/August, using Human Landing Catches (HLC) [24, 26]. Adult mosquitoes were collected from inside and outside houses, from 18 h00 to 06 h00 for seven consecutive nights in November, and from 17 h00 to 06 h00 for five consecutive nights in March and July/August. For each HLC house, a 2-person team collected mosquitoes from 18 h00 (or 17 h00) to 00 h00, and a second 2-person team collected from 00 h00 to 06 h00. One collector sampled indoors, sitting near the sleeping area of the inhabitants, and the second collector sampled outdoors, sitting about 2 to 5 m away from the house entrance. Each collection hour was comprised of a 50-min collection period and a 10-min break for the collectors. To reduce collection bias, the collectors switched collection position at the end of each collection hour. One supervisor per 2-person team verified quality of collections. HLCs were applied to obtain *Anopheles* landing rates inside and outside houses, as a proxy for human biting rate (HBR).

### Sample processing

At the end of the collection night, adult mosquitoes captured via HLCs were killed immediately with fumes of RAID (SC Johnson), a commercial aerosol insecticide for domestic use. Dead mosquitoes were then sorted to genus-level in the field based on morphological traits [27] and counted and recorded. Only *Anopheles* samples were retained for further processing and species identification. *Anopheles* were stored in Eppendorf tubes using silica gel and cotton wool. A random subsample underwent molecular analysis to confirm species identification. These results were published in Ávila et al. [14].

### Human behaviour observations (HBOs)

Via HBOs, HLC collectors documented intervention use and sleeping patterns in each of the two HLC houses in both sites [3]. At the end of each HLC collection hour, the HLC collector positioned outside the HLC house counted and recorded the number of people outside who were (1) asleep/awake (within about a 6-m radius), while the HLC collector positioned inside the HLC house counted and recorded the number of people inside who were (2) awake and not under a bed net, (3) asleep under bed net, and (4) asleep not under a bed net. However, in November, bed net use was not taken into account and collectors only recorded sleeping patterns of household members without considering bed net use. The HLC collectors were excluded from these HBO count data. The HLC collectors conducted these HBOs at the end of each of the 12 (for November and August/July collections) or 13 (for March collections) HLC collection hours. Data was verified by a supervisor and entered into an Excel spreadsheet.

### Ethical considerations

Both HLCs and HBO received ethical clearance by the National Committee on Bioethics of Research of Panama (CNBI) (EC-CNBI-2018-07-34). Before the start of collections in the communities, MINSA and implementing partners held meetings with community leaders of the selected sites to explain the purpose of the study and to obtain their authorization to work in the communities. Prior to enrollment of community volunteers into the collections, objectives and procedures were explained by MINSA health workers in the language of their choice, and signed informed consent were obtained from participants. To mitigate the risk of contracting malaria among participants, the MOH provided prophylaxis and Rapid Diagnostics Tests (RDTs) if needed during the sampling period.

### Data analysis

The HBR was calculated as the number of *Anopheles* biting per person, per location (inside/outside), per hour (bph) for a single night. To integrate the HBR with the HBO data, the HBO-adjusted HBR was calculated as in Monroe et al. [21]. Because bed net use was not included during the November collections, the March data on the proportion of people sleeping inside under a bed net in Permé and Puerto Obaldía was extrapolated and applied to the November dataset to estimate the use of bed nets in both sites in November 2018.

## Results

### *Anopheles* collections

In Permé, 7,245 *Anopheles* were collected in November (n = 3,833), March (n = 383), and August (n = 3,029). In Puerto Obaldía, 186 *Anopheles* were collected in November (n = 49), March (n = 66), and July (n = 71).

### *Anopheles* biting behaviour

**Fig. 2:**
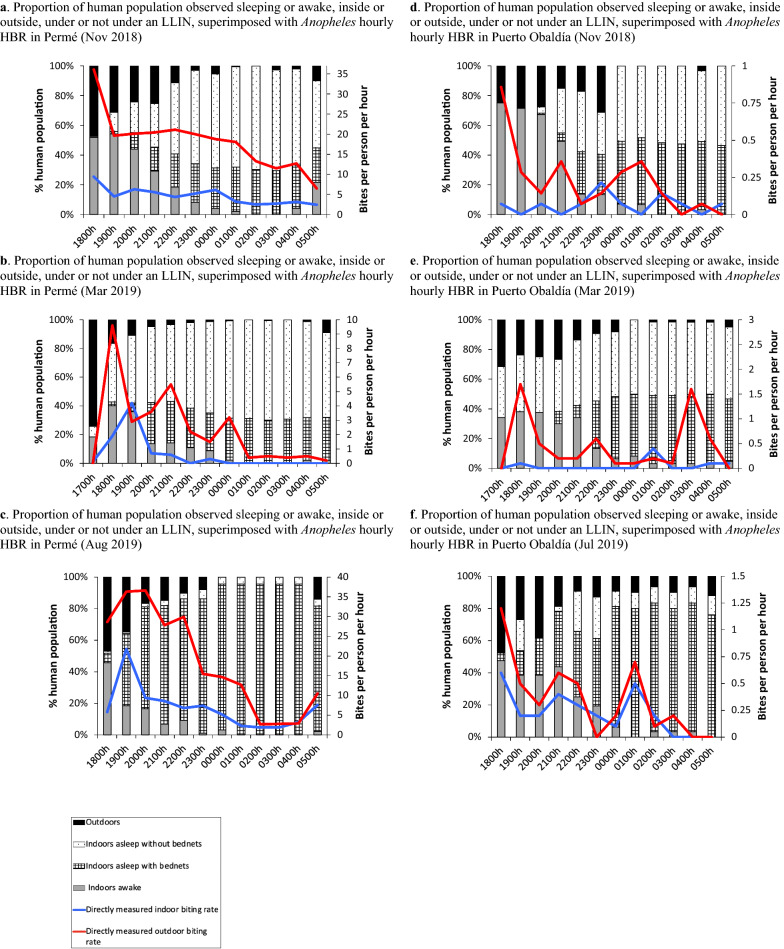
Proportion of human population observed sleeping or awake, inside or outside, under or not under an LLIN, superimposed with *Anopheles* hourly HBR from 18 h00 (Nov, Jul/Aug) or 17 h00 (Mar) to 06 h00, in Permé and Puerto Obaldía

Across the three sampling periods and both sampling sites, *Anopheles* biting activity was recorded throughout the night, both inside and outside, with an overall preference for outdoor biting (Fig. 2). In Permé, indoor landing rates fluctuated between 7.80 bites per person, per night (bpn) (March, dry season) and 81.90 bpn (August, heavy rainy season), while outdoor landing rates extended from 30.50 bpn (March), and 221.00 bpn (August). In Puerto Obaldía, indoor landing rates varied from a low of 0.70 bpn (March), to 2.70 bpn (July, heavy rainy season). In contrast, Puerto Obaldía’s outdoor landing rates ranged from 2.71 bpn (November) to 5.90 bpn (March). In general, peak indoor and outdoor biting activity in Permé and Puerto Obaldía occurred during the early evening hours (18 h00–19 h00) with secondary peaks in the later evening hours (20 h00–22 h00), before steadily declining until 6 h00 (Fig. 2).

### Human behaviour observations

Human behaviours around LLIN use and sleeping patterns inside and outside homes were examined alongside HLCs (Fig. 2). Across all three collection periods in Permé, most people were observed awake inside and outside up to 18 h/19 h, after which over 60% of people observed were then inside their homes (Fig. 2a–c). Most inhabitants went to sleep between 20 and 21 h, and several household members rose in the early morning hours (5–6 h). In March, LLIN use was very low (23%) (Fig. 2a, b); however, following the LLIN campaign in July 2019, LLIN use in August substantially increased (76%) (Fig. 2c). In Puerto Obaldía, inhabitants were observed awake inside and outside up to around 23 h when they went to sleep, and few rose before 6 h (Fig. 2d–f). In March, LLIN use was at 29% (Fig. 2d, e), but increased to 54% in July (Fig. 2f).

### HBO-adjusted HBRs

**Fig. 3:**
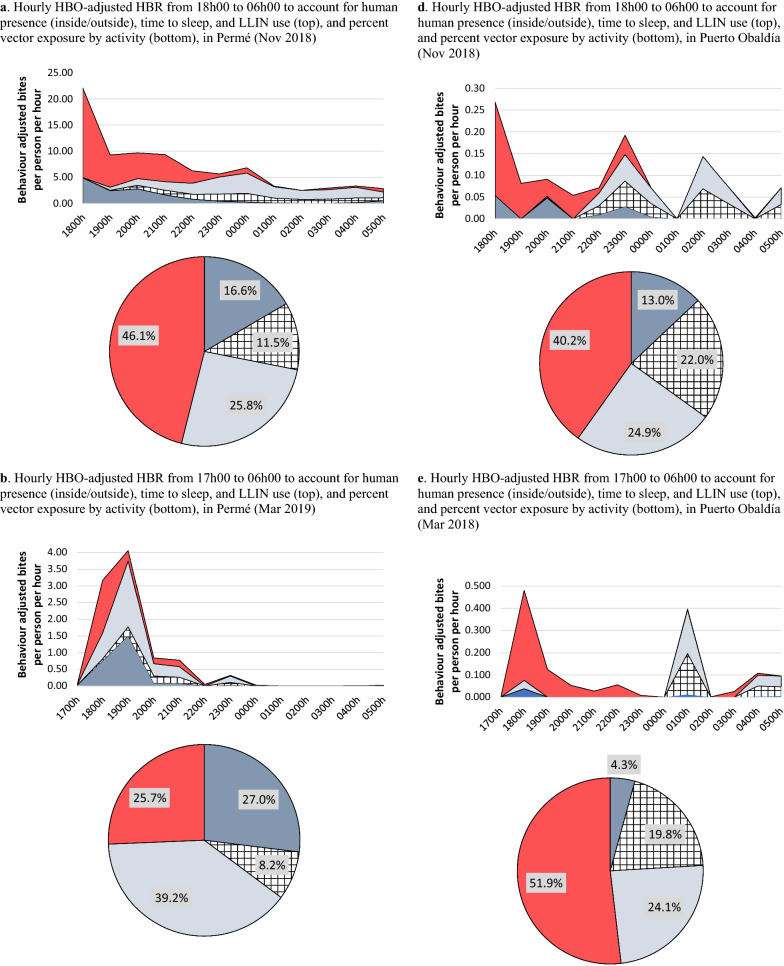

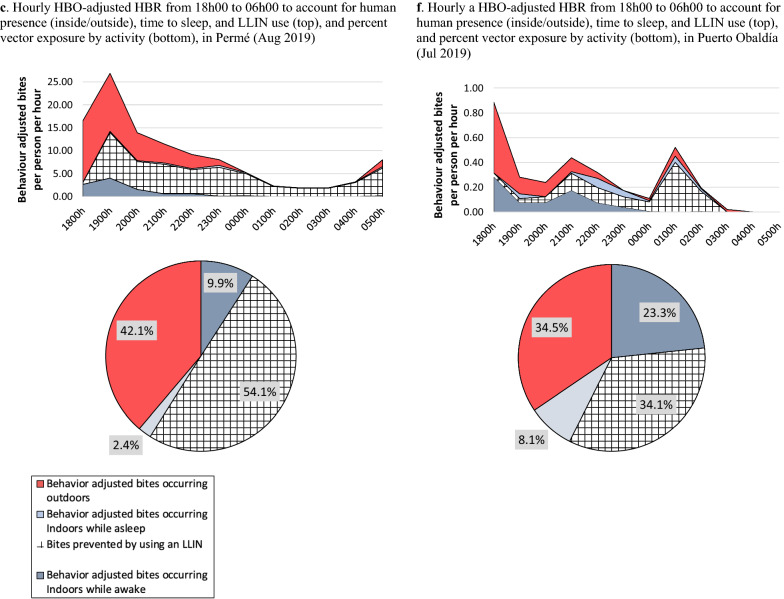
Hourly HBO-adjusted HBR from 18 h00 (Nov, Jul/Aug) or 17 h00 (Mar) to 06 h00 to account for human presence (inside/outside), time to sleep, and LLIN use, and percent vector exposure by activity, in Permé and Puerto Obaldía

Directly measured HBRs were adjusted to account for human presence (inside, outside), time inhabitants went to sleep, and LLIN use, i.e., HBO-adjusted HBR (Fig. 3). In November in Permé, indoor human-vector exposure was slightly lower (42%) than outdoor exposure (46%) (Fig. 3a); however, in March, indoor exposure increased to 66% (Fig. 3b). In November and March, sleeping without an LLIN accounted for 26% (Fig. 3a) and 39% (Fig. 3b) of the total potential human-vector exposure, and outdoor exposure occurred primarily from 17 h/18 h to 21 h, with a sharp decrease at 18 h/19 h when people went indoors for the night (Fig. 3a, b). However, in August, following an LLIN campaign, LLIN use increased, preventing 54% of exposure to vectors, resulting in outdoor vector exposure (42%) substantially exceeding indoor exposure (12%) throughout the night (Fig. 3c).

In Puerto Obaldía, outdoor exposure to *Anopheles* was higher than indoor exposure across all 3 collection periods. Outdoor exposure was predominant from 17 h/18 h to 23 h, after which indoor exposure slightly increased as people went inside for the night (Fig. 3d–f). Indoor vector exposure while asleep without LLINs accounted for about 25% of total potential exposure to biting in both November and March (Fig. 3d, e), but dropped to 8.1% in July (Fig. 3f).

## Discussion

Malaria transmission occurs when people are exposed to infectious vector bites because they are not adequately protected by the interventions in place, if any. In this operational investigation in Guna Yala, entomological collections in Permé and Puerto Obaldía were integrated with HBOs to assess the appropriateness of LLINs for reducing exposure to local *Anopheles*, and to identify and quantify remaining gaps in protection.

In Ávila et al. [14], the authors reported that the *Anopheles* species collected during this investigation included *Ny. albimanus*, *An. (Ny.) aquasalis*, *An. (An.) pseudopunctipennis s.l., An. (An.) punctimacula s.l.*, and *An. (An.) apicimacula*. As all are known vectors of *Plasmodium* [26, 28–31], genus-level data is sufficient for measuring both the genus-level biting trends (HBR), and for measuring the HBO-adjusted HBR towards quantifying human-vector exposure points. Overall, *Anopheles* HBRs remained substantially higher in Permé than in Puerto Obaldía, across all seasonal time points (Fig. 2). In low transmission settings such as Guna Yala, quantifying location-specific *Anopheles* landing rates is appropriate for estimating disease risk [32]. Hence, these findings indicate that Permé is likely more vulnerable to malaria than Puerto Obaldía. In fact, in 2019, Permé incurred more malaria than Puerto Obaldía: 20 cases for a total population of 155 in Permé, in contrast to 12 cases for a total population of 596 in Puerto Obaldía [14].

In both localities, *Anopheles* host-seeking activity was observed indoors and outdoors throughout the night. *Anopheles* landing rates were substantially more elevated outdoors than indoors, and usually higher earlier in the evening, than later in the night (Fig. 2) [14]. If MINSA had only considered these entomological data in their analysis and decision-making, then MINSA may have focused primarily on interventions that target outdoor biting in both sites. Yet, upon integration of *Anopheles* landing rates (i.e., HBR) with the HBOs, nuanced and contrasting human-vector exposure profiles were observed across each collection time point, and across both communities despite their proximity to each other.

In November, both outdoor and indoor vector exposures were higher in Permé (46% (outdoor), 42% (indoor)) than in Puerto Obaldía (40% (outdoor), 38% (indoor)). Elevated indoor vector exposure in Permé was attributed to low LLIN use in Permé (12%) compared to LLIN use in Puerto Obaldía (22%). Together with elevated outdoor exposure, this left Permé as the community most vulnerable to exposure to infectious *Anopheles* bites. However, in March, a notable shift in human-vector exposure profiles was observed: *outdoor* vector exposure became more prominent in Puerto Obaldía (52% of total potential vector exposure), while *indoor* exposure became more prominent in Permé (66% of total potential vector exposure). These opposing human-vector exposure profiles likely resulted from human behaviour differences rooted in distinctive lifestyle habits and cultural practices. Inhabitants of Puerto Obaldía spent more time socializing outdoors throughout the evening, while Permé inhabitants either spent more time indoors within their family units or went to sleep earlier in the evening. Absence of electricity in Permé likely explains this tendency to spend less time outside during the later evening hours and to go to sleep earlier in the evening. Thus, earlier sleep times increased the duration of opportunity for indoor vector exposure in Permé, which was compounded by notably lower LLIN use in Permé (8%) than in Puerto Obaldía (20%).

The integration of entomological and human behavioural data confirmed the relevance of LLINs to address indoor exposure to malaria vectors while asleep, and indicated a need to optimize this current intervention already in place [33] by increasing LLIN use in Guna Yala. These findings supported MINSA’s decision to continue with the LLIN campaign already underway in Guna Yala in July 2019, just before the third and final entomological sampling period. The campaign also included a social behaviour change communication (SBCC) strategy in an effort to improve LLIN use in the area. It should also be noted that due to logistical and financial constraints, this investigation did not conduct any insecticide susceptibility testing of the local vectors against the LLIN’s insecticides. Susceptibility to insecticides used in LLINs is key to ensure community protection [34], and any indication of insecticide resistance would be a gap in protection.

Following the July LLIN campaign in Permé, inhabitants of the community demonstrated a behavioural shift towards drastically increased LLIN use (Fig. 3c). During the July sampling period in In Puerto Obaldía, although the LLIN campaign had not yet been fully launched, LLIN use had also increased (Fig. 3f), perhaps because the SBCC materials recently distributed in preparation for the full LLIN campaign had already started to influence LLIN use behaviours in this community. By July/August, as increased LLIN use reduced overall indoor exposure, outdoor exposure became predominant over indoor exposure in both communities. The HBO-adjusted HBRs measured in this investigation demonstrate that it is critical to factor in human behavioural data when estimating the impact of interventions on human-vector exposure profiles, because it allows NMCPs to quantify the actual protection conferred by interventions in place and the remaining gaps in protection [21].

While this ESPT-based, MINSA-led operational investigation demonstrates that LLINs are a useful intervention to deploy in Guna Yala, this investigation also sheds light on the limitations of relying on a given intervention, such as LLINs, to reduce the malaria burden to zero. Interventions must be selected and appropriately deployed in response to changing vector behaviours [35]. Evaluating the HBO-adjusted HBRs pre- and post-LLIN campaign demonstrates how intervention deployment triggers shifts in human-vector exposure profiles, highlighting the non-static nature of human-vector dynamics [35, 36]. Entomological surveillance and control strategies that respond to these shifts are critical for NMCPs to deploy appropriate and effective interventions, and thus, to accelerate their progress towards elimination [37]. While MINSA further reduced indoor exposure by optimizing its LLIN campaign strategy in Guna Yala, there now remains two important gaps in protection: outdoor biting and indoor biting outside sleeping hours. Human-vector exposure that occurs outside the exposure points targeted by LLINs and IRS is a leading cause of persistent malaria transmission in malaria endemic countries [38, 39]. Additional interventions that can be used alongside LLINs and IRS and that confer additional community protection are required. The WHO Global Malaria Programme (GMP) recommends that in areas where outdoor transmission is occurring, there be a focus on evaluating the practicality, effectiveness, and affordability of novel control interventions [33]. For instance, larval source management (LSM) is well-accepted by the communities of Guna Yala [13], and in Colombia, nematode applications to target *Ny. albimanus* led to a decrease in larval density that was linked with a decline in malaria cases in children [40]. However, larval control is very labor-intensive, expensive, and logistically challenging, particularly in a densely forested region such as Guna Yala, where larval sites are abundant, cryptic, and ever-changing. Further, as LSM impacts malaria burden is poorly understood [41, 42], LSM is not likely a resource-effective intervention strategy for MINSA. Inversely, volatile pyrethroid-based spatial repellents are a highly promising new tool that are less laborious and more practical than LSM. Spatial repellents work by repelling outdoor biting vectors, and have demonstrated lethal effects on the impacted vectors [43–45]. To date, WHO has not established a position statement regarding the applications of spatial repellents in public health vector control, although it does recommend topical repellents for personal protection [46, 47]. Still, a recent landscaping analysis and review on repellents for mosquito control found clear consensus amongst the malaria community that spatial repellents have a place in vector control, while recognizing the need for evidence of epidemiological impact and a better understanding of how these interventions function [48]. In the ecological context of Guna Yala, Panamá, MINSA could consider piloting spatial repellents to target outdoor and early evening biting in the peri domestic area, as recent evidence indicates that spatial repellence has the potential to reduce malaria transmission [49]. Plus, as malaria cases are reported from Permé and Puerto Obaldía during the dry season, pertinent selection and sufficient intervention coverage throughout the dry season are also essential in both communities to protect community members from malaria [14].

Finally, due to logistical and financial constraints, this investigation did not conduct any insecticide susceptibility testing of the local vectors against the LLIN’s insecticides. Susceptibility to the insecticide used in the LLIN is key to ensure community protection.

## Conclusion

Malaria in Panamá persists only in small pockets across four malaria endemic regions. Therefore, MINSA must continue to strive to adopt a highly focalized and targeted approach to entomological surveillance and control to successfully address gaps in protection. The pronounced heterogeneity in HBO-adjusted HBRs across seasons and neighboring communities highlights the critical need for local data to guide decision-making for targeting and tailoring of vector control strategies. Further, the collection of local data based on priority programme questions helps assure the long-term sustainability of programmatic entomological surveillance and control. This ESPT-based operational investigation was conducted within the bounds set by programme capacity for entomological surveillance, and the collected entomological indicators were selected based on the programme question (appropriateness of LLIN based on vector bionomics and human behaviour) and on how these interventions function in relation to human behaviour towards intervention use. This framework enabled the programme to allocate their limited resources to the collection of minimum essential entomological indicators while ensuring the collection of meaningful data for their programme objectives.

## Data Availability

Data supporting the analysis, outcomes, and conclusions of this article are available upon request to the corresponding author.

## Abbreviations

GMP: Global Malaria Programme
HLC: Human landing catch
HBOs: Human behavior observations
IRS: Indoor residual spraying
LLINs: Long-lasting insecticidal nets
MINSA: Ministerio del Salud de Panamá
